# Health care providers’ perceptions of use and influence of clinical decision support reminders: qualitative study following a randomized trial to improve HPV vaccination rates

**DOI:** 10.1186/s12911-017-0521-6

**Published:** 2017-08-10

**Authors:** Brian E. Dixon, Monica L. Kasting, Shannon Wilson, Amit Kulkarni, Gregory D. Zimet, Stephen M. Downs

**Affiliations:** 1 Department of Epidemiology, Indiana University Richard M. Fairbanks School of Public Health, 1050 Wishard Blvd, RG5, Indianapolis, IN 46202 USA; 2 Regenstrief Institute, Inc., Center for Biomedical Informatics, 1101 W. 10th St, Indianapolis, IN 46202 USA; 3 Center for Health Information and Communication, Department of Veterans Affairs, Veterans Health Administration, Health Services Research and Development Service, 1481 W. 10th St, 11H, Indianapolis, IN 46202 USA; 4 Moffitt Cancer Center, Department of Health Outcomes and Behavior, 4115 East Fowler Ave, Tampa, FL 33617 USA; 5 Department of Pediatrics, Indiana University School of Medicine, 410 W 10th Street Suite 1001, Indianapolis, IN 46202 USA; 6 Merck & Co., 2000 Galloping Hill Road, Kenilworth, NJ 07033 USA

**Keywords:** Papillomavirus vaccines, Papillomaviridae, Immunization, Clinical decision support systems, Public health informatics

## Abstract

**Background:**

Human Papillomavirus (HPV) leads to serious health issues and remains the most common sexually transmitted infection. Despite availability of effective vaccines, HPV vaccination rates are suboptimal. Furthermore, providers recommend the HPV vaccine less than half the time for eligible patients. Prior informatics research has demonstrated the effectiveness of computer-based clinical decision support (CDS) in changing provider behavior, especially in the area of preventative services.

**Methods:**

Following a randomized clinical trial to test the effect of a CDS intervention on HPV vaccination rates, we conducted semi-structured interviews with health care providers to understand whether they noticed the CDS reminders and why providers did or did not respond to the prompts. Eighteen providers, a mix of medical doctors and nurse practitioners, were interviewed from five publicly-funded, urban health clinics. Interview data were qualitatively analyzed by two independent researchers using inductive content analysis.

**Results:**

While most providers recalled seeing the CDS reminders, few of them perceived the intervention as effective in changing their behavior. Providers stated many reasons for why they did not perceive a change in their behavior, yet the results of the trial showed HPV vaccination rates increased as a result of the intervention.

**Conclusions:**

CDS reminders may be effective at changing provider behavior even if providers perceive them to be of little use.

**Trial registration:**

ClinicalTrials.gov Identifier: NCT02551887

Registered on September 15, 2015

**Electronic supplementary material:**

The online version of this article (doi:10.1186/s12911-017-0521-6) contains supplementary material, which is available to authorized users.

## Background

Human Papillomavirus (HPV) is the most common sexually transmitted infection in the U.S., with approximately 79 million Americans already infected and 14 million new cases each year [1]. Infection with HPV is a causal factor for serious health issues including cervical cancer, anal cancer, penile cancer, oropharyngeal cancers, genital warts, and recurrent respiratory papillomatosis [2].

Despite the availability of a 9-valent HPV vaccine (9vHPV) that prevents up to 80–90% of cervical cancers and 90% of genital warts [3], HPV vaccination rates in the U.S. remain lower than desired to best protect the population against HPV infection [4]. In 2015, only 62.8% of adolescent girls and 49.8% of adolescent boys ages 13 through 17 years received one or more doses of vaccine [5]. The percentages are even lower for series completion (41.9% of girls and 28.1% of boys).

Existing research demonstrates that many physicians do not strongly endorse HPV vaccination or do not deliver timely recommendations [6, 7]. This is of particular concern because one of the strongest predictors of vaccine uptake is healthcare provider recommendation, and a lack of provider recommendation has been reported as a key reason for non-vaccination [8–12].

Computer-based clinical decision support (CDS), which provides appropriate, timely, patient-specific reminders and information to providers, can be effective at changing provider behavior. When implemented effectively, CDS has been shown to improve quality of care [13–17], and can be particularly effective for increasing appropriate use of evidence-based preventive services [14, 16, 18]. The federal “meaningful use” program for electronic health record (EHR) adoption in the U.S. further offers incentives for use of CDS [19]. The first stage of the meaningful use program required providers to adopt at least one CDS rule, and the second stage requires that they implement at least five CDS interventions that promote their institutional quality goals [20]. Later stages of the meaningful use program further encourage submission of information following vaccine administration to public health departments [21].

Researchers at Indiana University and the Regenstrief Institute possess a long history in using CDS to improve health outcomes and preventive services [22]. Pioneering studies in both inpatient and outpatient settings at Regenstrief provided CDS reminders to providers that encouraged the adoption of preventive services, including influenza and pneumococcal vaccination for eligible patients [23, 24]. In one study [24], over half of the 6371 patients admitted to a general medicine service during an 18-month period were eligible for one or more of the preventive CDS reminders. Provider ordering rates (intervention vs. control) were 35.8% vs. 0.8% for pneumococcal vaccination and 51.4% vs. 1.0% for influenza vaccination (*p* < 0.001 in both cases).

### Objectives of the study

Given poor HPV vaccination rates in Indiana and prior research on CDS system impact on provider behavior that increased vaccination for influenza and pneumococcal diseases, we designed a randomized clinical trial to test the effect of CDS on HPV vaccination. Like previous CDS efforts, our study offers the opportunity to learn about how providers respond to CDS prompts in the context of health care delivery.

A quantitative analysis of the trial is published elsewhere [25]. Another publication from our study examines provider awareness of the nine-valent form of the HPV vaccine approved for use at the beginning of our study [26]. Our team further published an examination of providers’ general perceptions of HPV vaccination and whether risk compensation influences recommendation of the vaccine to adolescent patients [27].

In this paper, we summarize the qualitative analysis of interviewers conducted after the trial in order to understand whether providers noticed the CDS reminders, which prompted providers to recommend HPV vaccination. We further examined why providers did or did not respond to the prompts (i.e. changed behavior).

### Study context

#### Setting

Eskenazi Health is one of the five largest safety net health systems in the United States. The health system contains a 315-bed hospital and nine community health centers located across the metropolitan area of Indianapolis, the eleventh largest city in the United States. There are five pediatric clinics among the nine health centers.

#### System details

The Child Health Improvement through Computer Automation system (CHICA) is an operational CDS system that has been used to support clinical practice for 12 years [28] and functions as a front end to an EHR system. When a child is registered in an Eskenazi pediatric clinic, the registration system sends an HL7 ADT (registration) message to CHICA. In response, CHICA queries a copy of the patient’s medical record from the EHR system. CHICA then applies hundreds of Arden Syntax rules to the data to select 20 structured, simple “Yes or No” questions that are displayed on an electronic tablet provided to the patient’s family in the clinic waiting room [29]. Family members answer the questions and return the tablet to a medical assistant who then enters the child’s height, weight and other measurements into the tablet. The information from the family and the medical assistant are then uploaded back into CHICA.

At the same time that CHICA produces the questions for the family to answer, the system sends an HL7 request to CHIRP (Children and Hoosier Immunization Registry Program), Indiana’s immunization information system or IIS [30, 31]. In response, CHICA receives a download of the child’s immunization history. The download includes CHIRP’s “forecast” of the immunizations for which the patient is due. The electronic transfer of immunization information between CHICA and CHIRP is a form of health information exchange (HIE) [32].

**Fig. 1:**
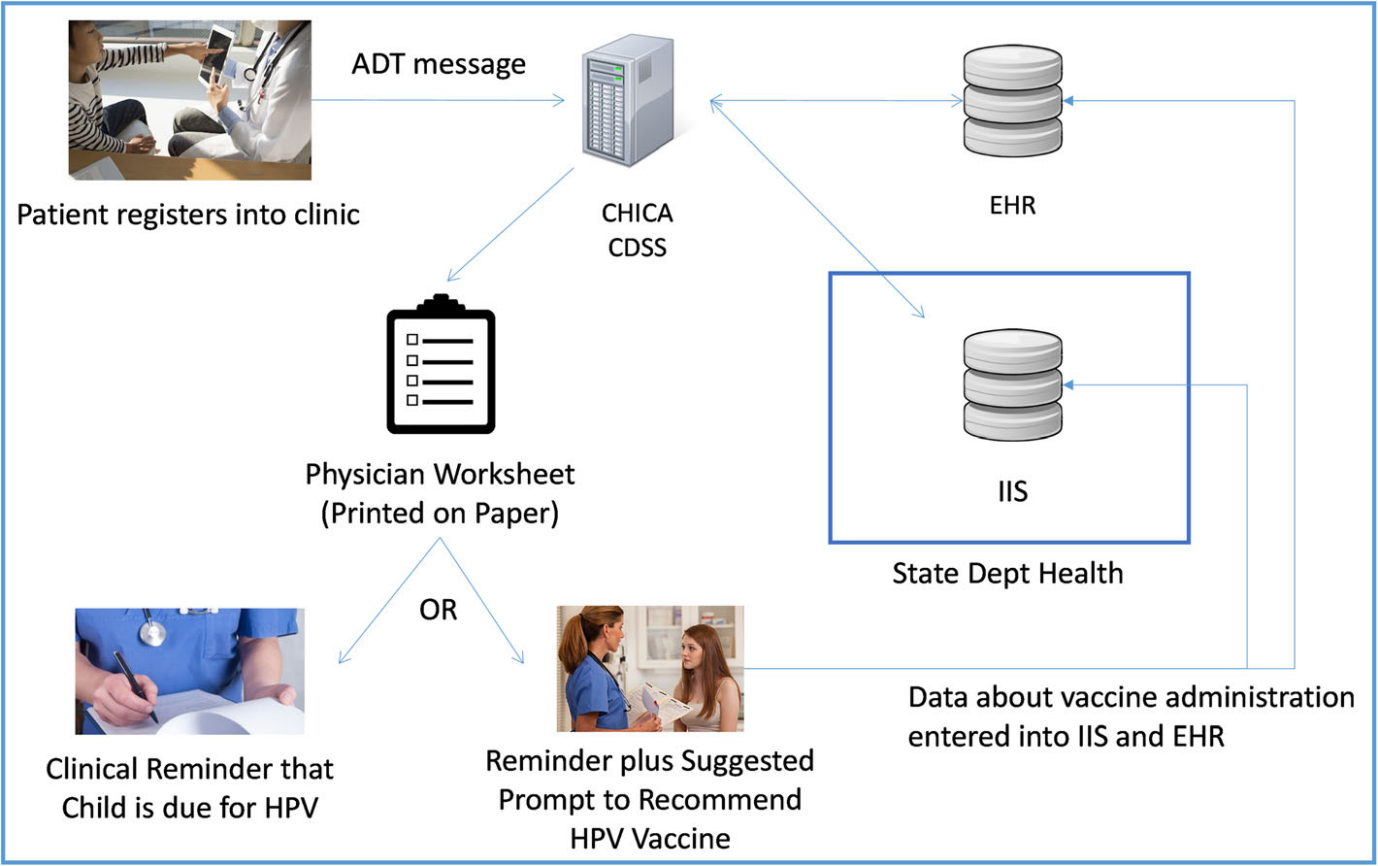
Technical architecture for how the CHICA clinical decision support system (CDSS) intervention communicates with the electronic health record (EHR) system at the clinic and immunization information system (IIS) at the state health department. Images used under license from Shutterstock.com with the Regenstrief Institute, Inc. ADT = Admission, Discharge and Transfer

At the end of both processes, CHICA produces several paper documents. The first is the physician worksheet. This worksheet includes up to six alerts and reminders for the provider. The reminders are selected by CHICA, using its Arden Syntax rule set, and are based on the patient’s EHR data as well as the data entered into the tablet. Each alert has up to six check boxes with which the physician can document how s/he responded to the alert. The physician worksheet, when completed, is scanned, and the coded data corresponding to the check boxes are stored along with any provider notes back to the child’s medical record. CHICA may also produce any of a large number of handouts for helping the provider with assessment or patient education. CHICA also produces a summary of the patient’s immunization history along with advice on shots for which the child is due [33]. The technical architecture is summarized in Fig. 1, which depicts the exchange of information between CHICA and the EHR as well as CHIRP, and the output of the process in the context of the randomized study.

## Methods

### Study design

To study CDS reminders for HPV vaccination, we conducted a randomized clinical trial (RCT) in which clinical reminders to recommend HPV vaccination were targeted to providers in pediatric outpatient clinics [25]. Randomization occurred at the level of provider. Providers were randomized to one of three arms: 1) usual care or the control arm in which no reminders for HPV were provided but the electronic health record (EHR) system was still used and CDS may prompt the providers regarding other vaccinations; 2) CDS reminders were provided to providers via the EHR system for HPV and other adolescent platform vaccines (prompt group); and 3) providers were provided both CDS reminders for vaccines plus a script to use when recommending the adolescent vaccines (prompt + script group).

The CDS reminders prompted providers to recommend the three adolescent platform vaccines: meningococcal (MCV4), HPV, and tetanus, diphtheria, and pertussis (Tdap) vaccines. The script provided to providers in the third arm read, “Three vaccines are recommended for <patient first name>, meningococcal to prevent meningitis, HPV to prevent cancer, and Tdap to prevent tetanus. All three are recommended at this age”. Eligible patients were children 11–14 years of age who had no previous history of HPV vaccination and no previous history of either MCV4 or Tdap. Eligibility was determined by querying both the EHR system (described below) and the state’s IIS (CHIRP) to establish a lack of prior vaccination history.

As a component of this larger RCT, we conducted semi-structured interviews with providers in each clinic between January and March 2015. The RCT and interview protocols were approved by the Indiana University Institutional Review Board. The primary, quantitative results of the RCT are published elsewhere. This article focuses on a qualitative analysis of interviews conducted with providers.

### Participants

Participants for this study were pediatric providers working in publicly-funded urban health clinics, had patients 11–14 years of age who were in need of vaccination, and consented to be interviewed. All eligible providers, medical doctors as well as nurse practitioners, were contacted via e-mail. Two additional follow-up e-mails were sent to each participant who did not respond to the initial e-mail. A total of 29 providers were eligible to be interviewed and 18 (62.1%) consented and completed the interview. Participants were recruited until saturation was reached, that is, until we acquired limited new information from the interviews [34].

### Interviews

Qualitative methodology is ideal when exploring an area where little is known, because it allows the investigators to identify, via in-depth analysis, relevant personal and contextual factors [35]. The majority of the interviews were conducted face-to-face (*n* = 16), but some were conducted over the phone if the provider could not meet in person (*n* = 2). All interviews were one-on-one and conducted by one of the co-authors (MLK). Interviews lasted 15–30 min, and participants were compensated with a $50 gift card.

After providing brief information regarding the study, all participants were asked about their general beliefs regarding HPV and HPV vaccination. Providers in the prompt and the prompt + script groups were then asked if they noticed the prompt and whether the prompt influenced their vaccination behavior. Providers in the prompt + script group were also asked if they noticed the script and if the script influenced their vaccination behavior or their conversations with the family. Participants in all three arms were asked if they discussed the CDS reminders and/or scripts with other providers in their clinic in order to assess contamination. Along with these questions, demographic characteristics (including sex, race/ethnicity, and years in practice) as reported during the interview were also collected. Discussions with participants were guided by a semi-structured interview guide [see Additional file 1].

### Data analysis

Interviews were audio-recorded, transcribed, and analyzed using inductive content analysis [36]. Content analysis is a qualitative research method that systematically and objectively describes phenomena [37, 38]. Research questions guide the identification of concepts, categories, themes, and conceptual models that are derived from qualitative data via insight and intuition of the researcher(s) [38]. Content analysis can be performed using induction or deduction techniques [39]. We employed inductive content analysis, which involves open coding of the transcripts, iterative generation of the categories or themes through discussion among the researchers, and summarization of the themes through narrative iterations.

Transcripts of the interviews were read by the authors to identify meaningful themes. Two investigators (MLK & SW) then independently coded each interview according to those themes. The codes were reviewed and areas of disagreement were resolved through discussion with the lead author (BED). Using the coded transcripts, we generated descriptive, summary information about each theme.

## Results

Here we present information on the respondents and the analysis of respondents’ comments. First, we present demographic information on the respondents. Next we present the key themes that emerged from analysis of the interview data.

### Respondent characteristics

Table 1 summarizes the demographic and participation data of the 18 participating providers. Most providers (*n* = 14) were women. Participants reported practicing medicine for a mean of 13.7 years (M = 10.5 years at their current clinic). The majority (*n* = 10) of providers were Caucasian, four identified themselves as African American, and four identified with ‘other.’ All arms of the trial were represented with slightly more providers (*n* = 8) from the CDS reminder plus script arm.

**Table 1:** Characteristics of participating pediatric healthcare providers

Characteristic	Count (%) or Mean *N* = 18	Standard deviation
Gender		
Male	4 (22%)	
Female	14 (78%)	
Race		
Caucasian	10 (56%)	
African American	4 (22%)	
Other	4 (22%)	
Years Practicing Medicine	13.7	8.1
Years in Current Clinic	10.5	5.1
RCT Study Arm		
Control	5 (28%)	
Reminder Only	5 (28%)	
Reminder plus Script	8 (44%)	

### Results of thematic analysis

Five major themes, summarized in Table 2, emerged from respondents’ comments. In response to specific questions during the interview, providers commented on both their awareness and utilization of the CDS reminders. When probed, providers commented on the reasons why they did not utilize the CDS reminders. The providers further described their perceptions of the effectiveness of the CDS reminders given their perceived use or non-use. Finally, providers suggested that their awareness of patient eligibility for a given vaccine is sometimes driven by information derived from nurses and not EHR systems.

**Table 2:** Themes and exemplar quotes from pediatric healthcare providers interviewed about CDS reminders that targeted provider recommendation of the HPV vaccine to eligible patients

Theme	Quotes
Awareness of CDS Reminders (Code: yes/no)^a^	“I’ve seen…I think I’ve seen…yeah I’ve seen the Menactra and the TDAP. I don’t know that I’ve seen HPV.” (no) “I believe so.” (yes) “Was I supposed to notice [a prompt]?” (no)
Utilization or Acceptance of CDS Reminders^a^	“Usually it will just help me remember to talk to the parents about it.” “I may just kind of briefly look at their shot record, but HPV is not one that I necessarily will focus on. So if I don’t happen to notice… I might not notice as often as if there was a prompt and I’m not sure if… Is there a prompt?” “I wouldn’t say necessarily they influence my vaccination practice.” “I didn’t respond negatively. I don’t mind it. I’m glad to have it, because sometimes it’s actually helpful for me.”
Reasons for non-use of CDS reminders^a^	“I didn’t change my behavior, like because it’s already drilled into our heads to just do the vaccines.” “I don’t think I did (notice the script) but maybe it’s because I think I’m doing it so you know, as we move through, I look and I see – you don’t have your three HPVs” “I was much better about it when we were charting on the paper....during the patient encounter that that’s just not a priority, unfortunately… It would be great if it were on the computer.” “So I am hoping when we don’t have the paper anymore when it is all tablet and it magically gets transported into our note and I can do that right at the beginning, I will read over that and it will prompt me, because truthfully I think I have really done not so good of a job of noticing the scary prompts.” “I don’t feel that I need CHICA to tell me to vaccinate because I like to vaccinate.” “Some percentage is not necessarily override, it’s just the amount of time I have to be able to cover all the topics.” “If it’s absolutely false, meaning if I know the family, especially if I know that they don’t have guns or things or that nature and they print out the gun sheet, then I don’t introduce that information to them.” “I do (use the prompts) when it works in my workflow. It is not that I don’t think they are helpful. It is just that as you know, in the flow of the day and the craziness of the day to go back and forth and remember to look at all the prompts to review for the concerning ones, that’s been difficult for me going to all of the laptop work.”
Effect of suggested script	“Oh, no, I don’t need help with that. I know what I need to say.” “Okay. It may have been there, but I’m being honest with you. I probably did delete it because I sort of have my own little spiel that I give.”
Role of nurses in vaccination	“[O]ur clinic, my nurse is just, she’s a hawk when it comes to vaccines.” “To be honest with you, the reason I usually know (the patient needs vaccines) is my nurse tells me. So she reviews the chart before I even see the patient and she’ll tell me they’re overdue.” “My nurse will be like, hey, it looks like they need this one, too, based on what CHICA’s thrown out, printed out.”

^a^Only asked of the providers in the CDS reminder groups

### Awareness and utilization of CDS reminders

Of the providers who received the CDS reminders (*n* = 13), those in the second and third arms, the majority (*n* = 9, 69%) indicated they noticed them. Many of these providers further held a positive view of the reminders, indicating that CDS reminders in general help them do their job. One provider noted, “If CHICA’s going to help me do my job, I think that that should be welcomed.” Yet several providers also stated that, while they held positive views of reminders, they did not believe the interventions changed their behavior, “…but I didn’t change my behavior, like because it’s already drilled into our heads to just do the vaccines.”

### Reasons for non-use of CDS reminders

Providers gave several reasons for not using, or ignoring, the clinical reminders. Many stated they were aware that all 11–12 year old patients are due for the adolescent platform vaccines and therefore do not need a reminder. When one provider was asked how she knew her patients were due for the HPV vaccine she responded, “Well, there are a couple ways. One is just realizing that usually around age 11 we vaccinate for [HPV].”

Providers noted that sometimes they do not look at the reminders, because they do not have time during a visit. As one provider stated, “I would say that sometimes we don’t have a chance to get to everything that it’s prompting, though, too, so sometimes—and there’s a list of maybe six, at least, items there, and it may happen in the visit that we ended up discussing other things for whatever reason.” Providers perceive they know what they are supposed to do at routine visits, and sometimes they will look at the reminders as a whole, after the patient leaves. For one particular physician, if they missed a prompt they deemed as “scary”, they will follow-up with a phone call to the patient. However, the prompts the providers deem as worthy of follow-up usually include those indicating imminent danger to the patient such as suicidal thoughts or food insecurities.

One of the most common reasons providers listed for not looking at the reminders was that the prompts are on paper and the patient chart is now on a computer, and switching back and forth between the two interrupted workflow. Many noted that it was easier to review the reminder sheets when the chart was also on paper but, now that they have transitioned to all electronic charts, the papers generally get ignored. One provider stated, “I don’t review them as much as I used to because we are more electronically oriented and carrying my laptop in the room… so the CHICA prompts are probably the last thing I might even look at.” Another said, “I was much better about it when we were charting on the paper because it was like right there but now honestly there are so many things that are printed out and everything that I need is on the computer in terms of the stuff I need to access during the visit.”

Providers also indicated that they have stopped paying close attention to the reminders because they are often incorrect or the parent misunderstood the prompt and answered incorrectly. They indicated that they know these families and have a rapport with them and can judge their risks better than the reminder system. One said, “I appreciate the prompts, but on the other hand, sometimes we know the patient better than the computer does.” Along the same lines, one provider recalled a prompt to discuss gun safety because there may be a gun in the home, but the provider said, “I know the family, especially if I know that they don’t have guns or things or that nature and they print out the gun sheet, then I don’t introduce that information to them.” Additionally, some providers said the parents do not understand the questions as they are presented on the tablet and may answer incorrectly, which gives the provider an inaccurate prompt. For example, one provider said, “I’ll ask about the prompts because it indicates the patient answered a certain way, but when I talk to the parent about it, they misunderstood the question or they didn’t think that that’s what they were answering and it doesn’t apply.” There is also the possibility that the parent is illiterate and cannot read the questions, or the parent may be comfortable answering a question on a tablet/computer but uncomfortable discussing it in-person with the provider and in front of their child. One provider mentioned this and said, “If you’re illiterate, some people may have issue saying, ‘I can’t read this’ or they may not want to say that in front of their kid.” Providers distrust prompts they receive consequently relying on their relationships and common practice to guide their encounters with their patients.

### Effect of suggested script for recommending HPV vaccine

Of the group that received both the CDS reminder and suggested script (*n* = 8), only one provider indicated he noticed the script. However, this provider indicated he did not use the script because, as he said, “I kind of don’t go with the script directly from that script to the parents. I’ve already discussed it in my way so I don’t really…I would say that that script that is in the CHICA form is not routinely used directly to be verbally said to the family.” Other providers said they did not notice it because they already have their “spiel” and know what they are going to say. When asked if they noticed or used the suggested script, one provider said, “Oh, no, I don’t need help with that. I know what I need to say.”

### Role of nurses in supporting vaccination processes

A final key theme was the effectiveness of nurses in reminding the provider that a patient is due for vaccination. When asked how he knew his patients were overdue for vaccines, one provider simply stated, “My nurse tells me.” Along those same lines, when asked about reminders for vaccination, 9 of the 18 providers specifically mentioned their nurse. One stated, “My nurse is just, she’s a hawk when it comes to vaccines.”

## Discussion

We interviewed 18 pediatric providers regarding their awareness, utilization and perceived effectiveness of CDS reminders implemented as part of an RCT designed to improve HPV vaccination rates. Among the providers who received the reminders, most reported noticing the CDS reminders or suggested language designed to improve how providers recommend HPV vaccination. Furthermore, respondents perceived the CDS reminders as useful but unlikely to influence vaccination rates given provider self-confidence in current performance and a variety of workflow issues associated with utilization of CDS reminders during a clinical encounter.

The contribution of this study is the detailing of reasons why providers did or did not accept CDS reminder and/or scripts provided to encourage HPV vaccination. These reasons suggest three important lessons for the broader biomedical informatics community, which includes developers, implementers and researchers. First, the use of qualitative methods to solicit feedback from providers is important to understand the impact of a health information technology system. Second, feedback from providers highlights the continued need for biomedical informatics system developers and implementers to focus their efforts on optimizing clinical workflow. Finally, the project demonstrates the potential for HIE, or integration between EHR and IIS systems, to improve population health. These results go beyond our prior examinations of pediatric providers’ general perceptions of the HPV vaccine and influences on whether or not they recommend the vaccine to adolescent patients [26, 27].

### Using mixed methods to understand mixed results

The quantitative analysis of the RCT, published elsewhere [25], found that the third arm of the study had a significantly higher rate of HPV vaccination than the control arm (62% vs 45%, *p* < 0.05), yet the rates of MCV4 and Tdap vaccination were equivalent across all three arms (MCV4: 81%, 81%, 83%; Tdap: 82%, 83%, 83%; *p* = 0.45 and 0.36, respectively). Furthermore, there was not a significant difference between the second and third arms (*p* > 0.1). The quantitative results suggest that the CDS intervention that included the recommendation script impacted HPV vaccination rates.

Methodologically, qualitative interviews with providers who participated in the RCT are useful in understanding attitudes and beliefs with respect to the CDS interventions in light of the mixed quantitative results. From the interview data, we observe only one provider in the third arm recalled noticing the HPV script, whereas many providers in the second arm recalled seeing the CDS reminders. Based on these data, it may be that the providers in the third arm noticed the reminder just not the suggested script, but the script may have nonetheless acted to enhance the salience of the prompt.

Although the HPV vaccination rate in the third arm was higher than in the first, providers perceived that the intervention had no effect on their behavior. While possible that providers in the third arm are better vaccinators than the providers in the first arm, the chance of this statistically is very low given the quantitative analysis. Therefore the discordance between the two sets of results suggest that in some cases CDS reminders may provide subliminal, unconscious messages that actually do affect behavior, even if the behavior change is not perceived by the provider. This conclusion, and the observations in this study, bolster prior research in which the researchers turned off CDS reminders to find that preventive tasks such as vaccination went down in the absence of CDS [22, 40].

### Workflow is important for provider adoption and satisfaction

Although CDS reminders can be effective, even when subliminal, informaticians must pay attention to clinical workflow. Respondents in this study commented on multiple aspects in which CHICA and the CDS reminders for HPV vaccination may not have fully supported the busy workflow or cognitive processes of pediatric providers. One issue is that the clinics with the HPV reminder intervention recently implemented EHR systems for clinical documentation, yet CHICA remained reliant on paper-based processes. When CHICA was introduced more than a decade ago, most of the clinical workflow relied on paper-based processes. However, with the recent transition to a greater number of EHR components implemented to comply with ‘meaningful use’ regulations [41, 42], provider workflows increasingly rely on computers for data capture, information retrieval and review of the patient’s chart. This created a cognitive challenge for providers who had to manage both paper and electronic information.

In addition, several providers indicated they often do not have time to review the CDS reminders during a patient encounter. These comments are emblematic of the complexity involved in EHR and CDS systems in a world where computers are integrated into workflow. A study by McDonald et al. [43] demonstrated that the introduction of EHR systems can increase the amount of time providers work each day due to the complexity of completing documentation in the EHR as opposed to on paper. Providers are also sensitive to the issue of ‘alert fatigue’ where CDS reminders can pose ‘one more thing’ they have to comply with in the age of EHR systems [44]. However, it should be noted this phenomenon was only mentioned by a couple providers so did not appear to be as much of an issue in this study as the disconnect between the EHR system and the paper-based CDS reminders.

A final item related to workflow involves the role of nurses in both information retrieval and the vaccination process. Several providers commented that their awareness of a patient’s vaccination history relies principally on the nurses who support care delivery within the clinic, which may explain why some providers reported not attending to CDS prompts. Nurses are often tasked with retrieving information from the patient’s chart, including tasks that access an IIS to forecast vaccines for which a child is due. Nurses typically then administer the vaccine under direction from a physician following patient (or family) consent. Given a general shift in medicine towards team-based care where each provider works ‘to the top of their license’ [45], it may be advantageous for CDS systems to target nurses as the recipients of reminders rather than, or in addition to, physicians. Furthermore, prior studies on CDS reminders in the inpatient setting show that standing orders for influenza and pneumococcal vaccines, in which nurses have the clinical authority to not only check on a patient’s eligibility for these vaccines but also offer and administer them without specific direction from a physician, can increase vaccination rates significantly more than CDS reminders presented to physicians [46]. Future studies on CDS reminders for vaccines, including HPV, in pediatric as well as family medicine clinics should explore CDS paradigms in which standing orders for non-physicians are compared with traditional clinical reminders. Additionally, researchers should consider CDS modalities that could provide reminders or prompts directly to patients (or guardians) prior to or during a clinic visit [47, 48].

### The role of health information exchange

Although the emphasis of this study focused on provider interaction with the CDS reminders, this study demonstrates the potential for HIE-based CDS systems to improve population health [49]. In the background, between the time the patient was registered into the clinic and when he or she was seen by a provider, the CHICA system integrated data retrieved from the patient’s EHR and the IIS at the state health department. Without HIE between CHICA and the IIS, nurses or other clinic staff would be required to directly access the IIS to retrieve information on the child’s vaccine history and forecast then manually reconcile this information with data in the EHR. This is how it works in most clinics within Indiana and likely the rest of the U.S. Interoperability and HIE [32] support electronic communication between disparate health information technology systems to support clinical workflow. Recent studies by others show that greater interoperability between EHR systems and IIS are possible as providers continue to adopt EHR systems for ‘meaningful use’ [21]. Future studies should more directly evaluate HIE and its impact on vaccine rates as well as other population health outcomes given greater access to integrated information at the point of care.

### Limitations

No study is without limitations. First, the study was conducted within a single, publicly-funded urban health system. Providers’ comments may not represent what might happen if a similar system was implemented elsewhere. Additionally, participants represent a purposive sample, in which the investigators sought to balance inclusion from all arms of the RCT. Yet respondents may have agreed to participate because they held either strongly positive or strongly negative views with respect to HPV vaccination or the CHICA system. To mitigate the potential for bias, we included opposing viewpoints expressed by respondents in the results where documented.

## Conclusion

While our trial of CDS reminders that targeted pediatric providers improved HPV vaccination rates, when asked about the intervention providers reported being aware of the prompts but did not believe they influenced their behavior. Providers believed they generally do a good job of vaccinating adolescents and that awareness of patient eligibility for a vaccine is influenced by other clinical team members’ situational awareness. These perceptions may influence some healthcare organizations to disable CDS reminders for vaccination even in the face of evidence that CDS can improve vaccination rates. Such an action is not recommended and would be counterproductive, even though the exact role of CDS reminders in changing provider behavior remains unclear.

Given this study, more research is necessary to further unpack the role of CDS in vaccination processes within clinics. One potential pathway may be to use CDS prompts that target other members of the clinical team, or there may be better ways to present reminders to providers in the context of clinical workflow. Pathways involving CDS direct to consumers should also be considered. Biomedical informatics researchers and innovators, including those who develop commercial systems, should focus time and effort on understanding clinical workflows and cognitive processes across health systems to design systems that can meet varied clinical practices. While helpful in advancing our understanding CDS, this study should be replicated and analyzed in other contexts to more fully account for the variety of practices and cognitive processes that underlie clinical decisions with respect to vaccination.

## Additional file

Additional file 1: Semi-Structured Interview Guide. (DOCX 22 kb)

## Abbreviations

CDS: Clinical decision support
CHICA: Child Health Improvement through Computer Automation system
EHR: Electronic health record system
HIE: Health information exchange
HPV: Human papillomavirus
IIS: Immunization information system
RCT: Randomized controlled trial
