# Supplementary material for: Health care providers’ perceptions of use and influence of clinical decision support reminders: qualitative study following a randomized trial to improve HPV vaccination rates
Source: BMC Med Inform Decis Mak. 2017 Aug 10;17:119. doi: 10.1186/s12911-017-0521-6 (PMC5553598; doi:10.1186/s12911-017-0521-6)
Supplement: Additional file 1: — Semi-Structured Interview Guide. (DOCX 22 kb) [file 12911_2017_521_MOESM1_ESM.docx]

Semi-Structured Interview Guide

HPV Vaccination: An Investigation of Physician Reminders and Recommendation Scripts

**Interview Questions**

**HPV Knowledge and Attitudes**

Let’s begin by discussing HPV vaccination in general. As you know, ACIP recommends HPV vaccination for all males and females 9-21 years of age.

1. Think about your last patient encounter in which a child between the ages of 11-12 was overdue for vaccines, such as Meningococcal, HPV, and/or Tdap…How did you know the child was overdue for one or more vaccines?

Potential Probes

- Was the information about vaccination on paper or the computer?
- Did you access CHIRP – the state immunization registry?
- Did you access the information via the EHR?

1. How did the conversation about vaccination go with the child and/or the family?

Potential Probes

- Was there any hesitancy on the part of the parent or child? How did they communicate hesitancy?
- What kind of concerns about vaccination did the parent or child tell you about?

1. In the past, some health care providers have expressed discomfort when vaccinating children age 12 and younger for the HPV vaccine. Do you have any reservations about discussing and/or offering the HPV vaccine with parents of and patients under 12?

Potential Probes

- Are you comfortable speaking with parents about the risks and benefits of the HPV vaccine?
- How often does sexual activity come up in your conversations with parents? The child?

| Possible Barriers to Ask About |
| --- |
| Uncomfortable conversation with parents of pre-teens regarding sexual behavior |
| Fear of parents who are anti-vaccination |
|  |
|  |
|  |

**CHICA Knowledge and Attitudes**

Clinical decision support (CDS) systems play an increasing role in supporting decision-making processes. We would therefore like to ask you a few questions about CDS system in your clinic, known as CHICA.

1. When do you typically review the prompts and guidance provided by CHICA?
2. Do you follow the prompts/alerts provided by CHICA?

Potential Probes:

- Under what circumstances do you override an alert? Why?
- Under what circumstances do you follow the guidance provided by CHICA? Why?

| Possible Barriers to Ask About |
| --- |
| Alert fatigue |
| Erroneous recommendations |

**FOR PROMPT & SCRIPT GROUPS ONLY:**

**Prompt group & Prompt + Script group:**

6. Did you notice the appearance of a CHICA prompt this past year for the 3 adolescent platform vaccines: Meningococcal Vaccine, Tdap, and HPV Vaccine?

7. How did you respond to the CHICA reminder prompts about these vaccines?

Potential Probes:

- Did you find the prompts to be helpful?
- Do you think your vaccination rates for HPV vaccine increased as a result of the reminder prompts?

**Prompt + Script group ONLY:**

8. Did you notice the suggested recommendation script on the CHICA sheet? (“Three vaccines are recommended for your patient, meningococcal to prevent meningitis, HPV to prevent cancer, and Tdap to prevent tetanus. All three are recommended at this age”).

9. How did you respond to the recommended script on the CHICA sheet?

Potential Probes:

- Did you find it helpful? If so, in what way?
- Did the recommended script change the way you talked about vaccines? If so, how?

**Assessment of Contamination**

10a. (Prompt Group Only): Have you discussed the CHICA reminders for HPV vaccines with your colleagues or ancillary staff? If so, what did you say?

10b. (Prompt + Script Group Only): Have you discussed the CHICA reminders or recommended script for HPV vaccines with your colleagues or ancillary staff? If so, what did you say?

10c. (Control Group Only): Were you aware of HPV vaccine prompts and a suggested recommendation script delivered via CHICA to other providers?

**Risk Compensation Questions**

It has been suggested that getting vaccinated against HPV could lead a young adolescent to engage in riskier sexual behavior because they might believe they are protected from the negative consequences of those actions.

What are your thoughts about this issue?

As a provider, do you believe HPV vaccine could lead your patients to practice riskier sexual behaviors in the future? If yes, why? If no, why not?

If yes, does this concern have any influence on your HPV vaccination practices? [who/when you vaccinate?]

Some people wonder if a young woman who is vaccinated against HPV might be less inclined to get screened for cervical cancer as an adult.

What are your thoughts about this issue?

Do you believe the HPV vaccine could result in your patients feeling protected from cervical cancer and they are therefore less likely to get screened for cervical cancer in the future? If yes, why? If no, why not?

If yes, does this concern have any influence on your HPV vaccination practices? [who/when you vaccinate?]

Do you discuss the importance of cervical cancer screening in the future regardless of HPV vaccination status?

**Demographic Questions**

Thank you for your time today. We’ve covered the main questions relevant to the study. However, before we end I have a few demographic questions if you don’t mind.

11. How many years have you been practicing medicine? _____

12. How many years have you practiced in your current clinic? _____

13. How would you describe your race? ________________

14. How would you describe your ethnicity? ________________

15. How would you describe your gender? ________________

**9-Valent Vaccine Questions**

Have you heard about the new 9-valent HPV vaccine?

If yes, did you hear that it was licensed by the FDA in December?

[Provide information to all: the new 9-valent vaccine is like Gardasil, but it includes protection against 5 additional cancer-causing HPV types. Gardasil protects against types 6, 11, 16, and 18. The new vaccine, called Gardasil 9, protects against these 4, plus types 31, 33, 45, 52, and 58. Gardasil 9 has the potential to prevent 90% of cervical cancers. The FDA approved Gardasil 9 for use in girls and young women 9 through 26 years of age and in boys 9 through 15 years of age.

What questions or concerns do you have about this new vaccine?
